# Supplementary material for: Effectiveness and Safety Profiles of Biological Therapies in Inflammatory Bowel Disease: Real Life Data from an Active Pharmacovigilance Project
Source: Biomedicines. 2022 Dec 18;10(12):3280. doi: 10.3390/biomedicines10123280 (PMC9775949; doi:10.3390/biomedicines10123280)
Supplement: Supplementary file 1 [file biomedicines-10-03280-s001.zip › biomedicines-2091301-supplementary.pdf]

Table S1. Total years of treatment and number of failures and adverse drug reactions divided by therapy-lines.

	1° line	Total years of treatment	Failure (Failure/10 yrs)	ADR (ADRs/10 yrs)	2° line	Total years of treatment	Failure (Failure/10 yrs)	ADR (ADRs/10 yrs)	3° line	Total years of treatment	Failure (Failure/10 yrs)	ADR (ADRs/10 yrs)
IFX	209	590.5	40 (0.7)	73 (1.2)	62	110.1	13 (1.2)	19 (1.7)	6	10.2	1 (1.0)	3 (2.9)
ADA	210	616.2	52 (0.8)	64 (1.0)	72	263.8	26 (1.0)	19 (0.7)	10	10.1	2 (2.0)	1 (1.0)
GOL					33	53.9	14 (2.6)	6 (1.1)	17	7.3	8 (11.0)	1 (1.4)
VED	7	3.2	2 (6.3)	2 (6.3)	96	152.3	28 (1.8)	15 (1.0)	112	180.5	30 (1.7)	22 (1.2)
UST					59	46.5	7 (1.5)	8 (1.7)	59	43.8	4 (0.9)	16 (3.7)

ADA= adalimumab; ADR= adverse drug reaction; GOL= golimumab; IFX= infliximab; VED= vedolizumab; UST= ustekinumab; yrs= years.

Figure S1. Kaplan-Meyer curve for the persistence of biologics as first-line therapy (a) and second- and third-line therapies (b).

a)

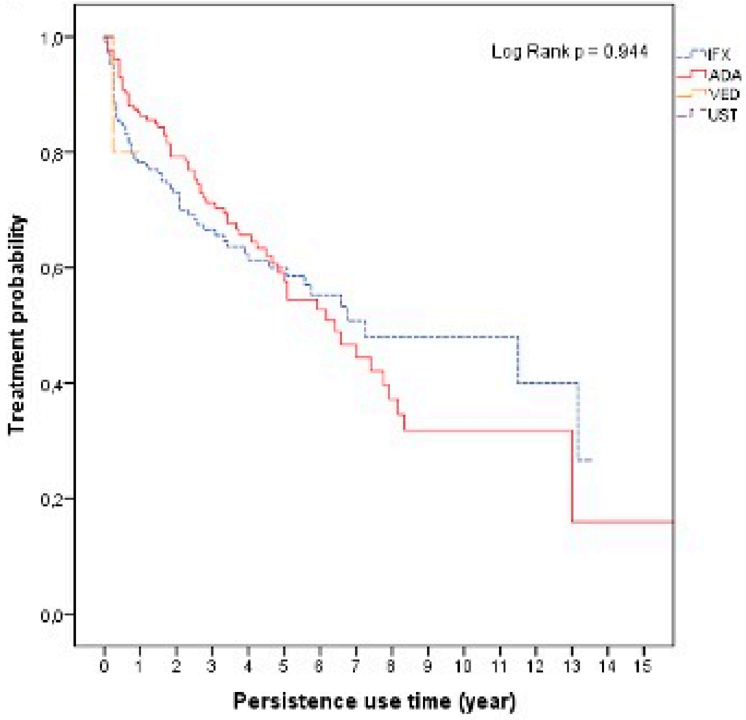

b)

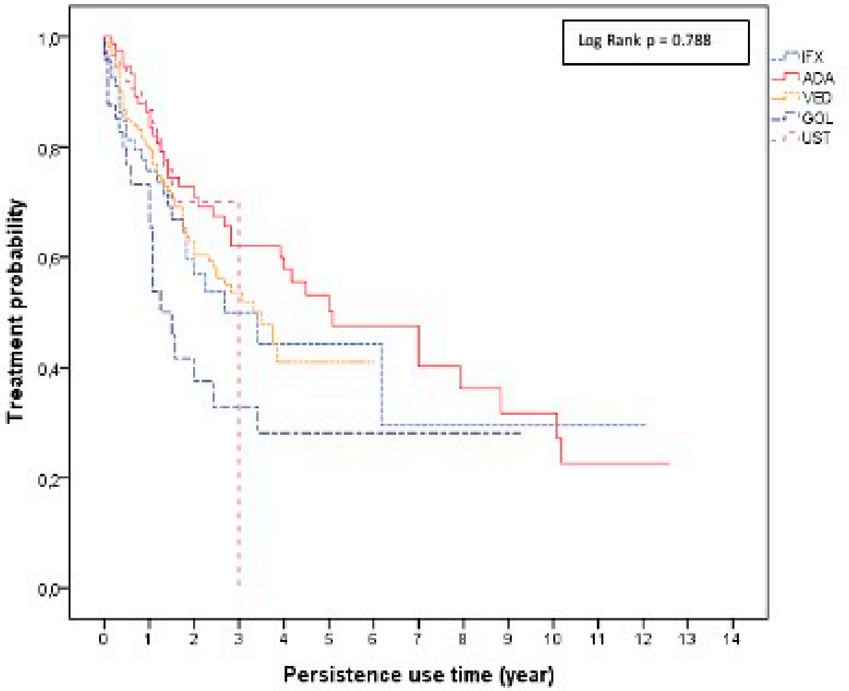

	Persistence Rate (%)					Median persistence time (95% CI)
	Year 1	Year 2	Year 3	Year 4	Year 5	
IFX	77.6	69.9	65.6	61.3	58.6	-
ADA	86.3	78.5	70.3	64.5	57.6	-
VED	-	-	-	-	-	-
UST	-	-	-	-	-	-
Overall	82.2	74.6	67.9	63.3	58.8	6.58 (5.33-7.83)

	Persistence Rate (%)					Median persistence time (95% CI)
	Year 1	Year 2	Year 3	Year 4	Year 5	
IFX	73.5	56.9	44.4	35.7	29.6	2.67 (0.94-4.40)
ADA	83.5	71.0	62.0	57.9	50.3	5.08 (2.53-7.63)
VED	79.4	60.3	51.9	41.0	-	3.50 (2.49-4.52)
GOL	65.4	37.5	28.1	-	-	1.50 (1.01-1.95)
UST	84.3	-	-	-	-	-
Overall	79.8	61.0	53.2	46.0	42.1	3.50 (2.61-4.39)

Kaplan-Meier curve for persistence of biologics among patients in a) first-line and b) second- and third-line therapy. If event rate is lower than 50%, the median survival or its 95% CI cannot be calculated.

Figure S2. Kaplan-Meyer curve for the persistence of biologics in patients with CD (a) and UC (b)

a)

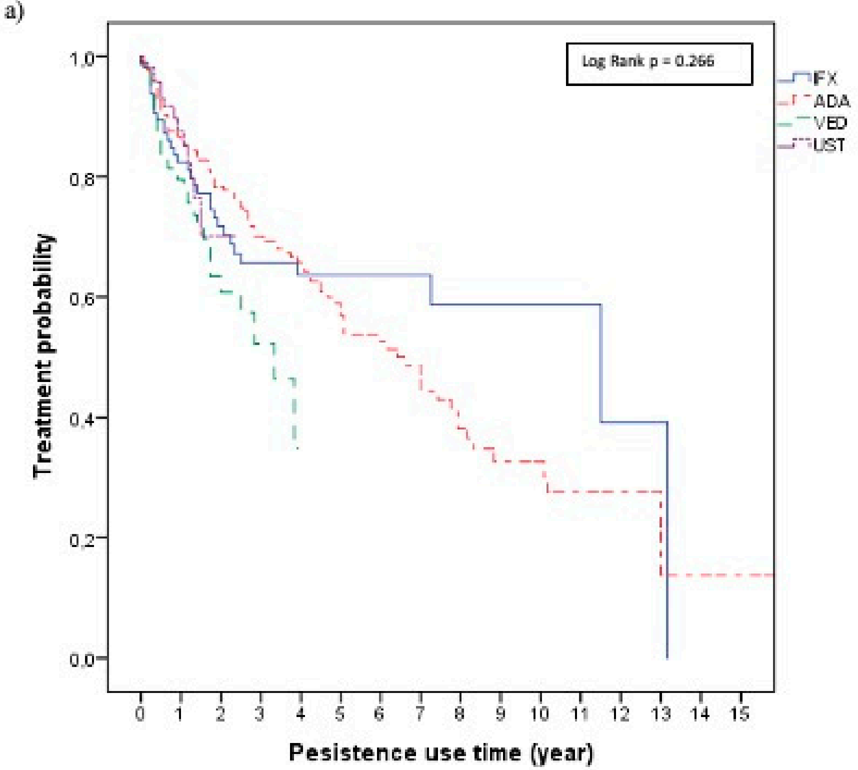

b)

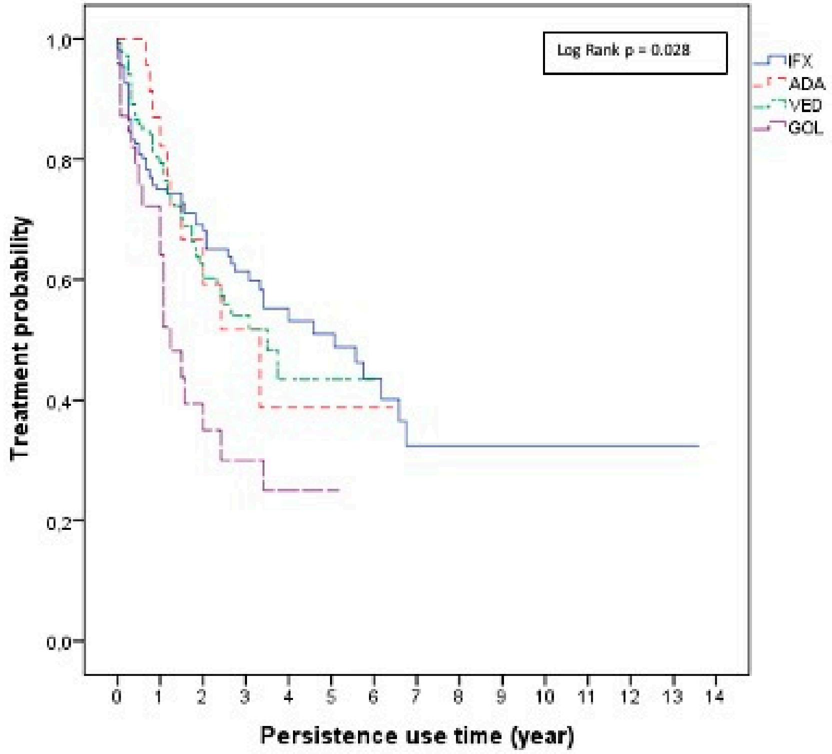

	Persistence Rate (%)					Median persistence time (95% CI)
	Year 1	Year 2	Year 3	Year 4	Year 5	
IFX	81.1	70.3	65.4	63.6	62.6	-
ADA	85.2	77.8	69.3	65.1	56.8	-
VED	77.6	60.8	46.4	34.8	-	-
UST	85.1	-	-	-	-	-
Overall	84.7	73.9	65.9	61.6	56.0	7.00 (5.36-8.64)

	Persistence Rate (%)					Median persistence time (95% CI)
	Year 1	Year 2	Year 3	Year 4	Year 5	
IFX	74.3	68.2	59.9	53.1	48.8	5.08 (2.92-7.24)
ADA	82.1	59.2	38.8	-	-	3.33 (1.47-5.19)
VED	79.3	60.2	51.7	-	-	3.50 (2.27-4.73)
GOL	64.1	35.0	25.0	-	-	2.86 (0.80-1.70)
Overall	76.0	61.8	54.6	46.1	43.1	3.42 (2.25-4.59)

Kaplan-Meier curve for persistence of biologics among patients with a) CD and b) UC. If event rate is lower than 50%, the median survival or its 95% CI cannot be calculated.

Table S2. Type of adverse drug reactions observed during the follow-up period by therapy lines.

Type of ADR, *n* (incidence per 10 yrs of treatment)	IFX First line	IFX Second line	IFX Third line	ADA First line	ADA Second line	ADA Third line	GOL Second line	GOL Third line	VED First line	VED Second line	VED Third line	UST Second line	UST Third line
SADRs*	12 (0.2)	4 (0.4)	1 (1.0)	13 (0.2)	9 (0.3)		3 (0.6)	1 (1.4)		4 (0.3)	12 (0.7)	4 (0.9)	2 (0.5)
Infections	18 (0.3)	3 (0.3)	1 (1.0)	17 (0.3)	8 (0.3)	1 (1.0)	2 (0.4)	2 (2.7)	1 (3.1)	7 (0.5)	5 (0.3)	1 (0.2)	9 (2.1)
Pneumonia				2 (0.03)	2 (0.1)					1 (0.1)	1 (0.1)	1 (0.2)	
Infusion/injection related reaction	23 (0.4)	13 (1.2)		3 (0.05)						1 (0.1)		1 (0.2)	1 (0.2)
Skin reaction	10 (0.2)			20 (0.3)	4 (0.2)		1 (0.2)					1 (0.2)	2 (0.5)
Joint disease	6 (0.1)	1 (0.1)		2 (0.03)	1 (0.04)						2 (0.1)		
Perianal disease	2 (0.03)	1 (0.1)	1 (1.0)	2 (0.03)	1 (0.04)						3 (0.2)		1 (0.2)
Malignancies	1 (0.02)		1 (1.0)	2 (0.03)	1 (0.04)		1 (0.2)			2 (0.1)	6 (0.3)		
Blood disorders	2 (0.03)			2 (0.03)	1 (0.04)					1 (0.1)			
Others	10 (0.2)	1 (0.1)		14 (0.2)	1 (0.04)		1 (0.2)		1 (3.1)	3 (0.2)	6 (0.3)	3 (0.6)	3 (0.7)

*SADRs include hospitalization, life-threatening, surgery, malignancies and death

ADA= adalimumab; ADR= adverse drug reaction; GOL= golimumab; IFX= infliximab; VED= vedolizumab; UST= ustekinumab; yrs= years.
